# Work ability literacy among managers in the health and social care sector – a qualitative study

**DOI:** 10.3389/ijph.2026.1610014

**Published:** 2026-09-22

**Authors:** Eveliina Korkiakangas, Maria Paavolainen, Kirsikka Selander, Risto Nikunlaakso, Maarit Kaartinen, Tiina Koivisto, Nina Nevanperä, Jaana Laitinen

**Affiliations:** 1 Finnish Institute of Occupational Health, Helsinki, Finland; 2 Aalto-yliopisto, Aalto, Finland

**Keywords:** management, managers, work, work ability, work ability literacy

## Abstract

**Objective:**

to deepen the understanding of the contents of work ability literacy among managers in the health and social care sector regarding both their own and their employees’ work ability.

**Methods:**

Semi-structured interviews were conducted from June to October in 2024 with 49 managers in the health and social care sector in Finland.

**Results:**

Managers’ knowledge about work ability and its promotion is shaped by organizational information, personal experience and education, and interactions within the work community. Their aim in supporting work ability is to ensure effective organizational operations, promote employee wellbeing, and fulfil their managerial responsibilities. Managers have varied understandings of work ability, which can focus on individual traits, personal life or adaptation and modification of work. Practical methods to support work ability include adjusting work content, referring employees to occupational health services, offering social support, enhancing professional skills, and promoting healthy lifestyles and recovery from work.

**Conclusion:**

To effectively support work ability, managers need adequate resources and more education on contents of work ability literacy. Especially skills and possibilities to prevent work disability are needed.

## Introduction

Finland’s government-funded twenty-one wellbeing services counties are responsible for primary and specialized health and social services (HSS), and rescue services [1, 2], employing over 220,000 professionals. The shortage of HSS professionals is a global challenge [3], also in Finland [4, 5]. Workforce shortages are driven by large-scale retirements, declining entry into the profession, and reduced work ability: 35% of HSS workers report decreased work ability, and only 39% feel they recover well from work [6]. These trends highlight the need to promote HSS personnel’s work ability.

The concept of work ability literacy was first defined as understanding of the demands and the effects of work on one’s health and work ability and actions one takes considering these demands and effects to promote one’s own work ability. The concept was tested among occupational health nurses in two contexts: how they understood the meaning of work ability literacy relating to their own and to their client’s work ability. Work ability literacy involved recognizing factors affecting work ability and supporting both one’s own and clients’ work ability, including facilitating positive change. Work ability literacy can be used as a concept or framework in the promotion of work ability, and it includes health-related knowledge, attitudes, motivation, behavioral intentions, personal skills, and self-efficacy, and the promotion of behavior change in client work [7].

Work ability literacy was based on the concepts of work ability and health literacy. Work ability is described here through the Work Ability House model, which presents the key factors influencing work ability. In this model, work ability is understood as a balance between individual resources—such as health, occupational competence, values, attitudes and motivation—and work-related factors, including work demands and tasks, the work community, leadership and management [7]. Health literacy refers to the ability of individuals to access, understand, appraise and use information and services in ways that promote and maintain good health and wellbeing for themselves and those around them [7, 8]. A definition of health literacy entails knowledge, motivation and competences to access, understand, appraise, and apply health information to make judgments and decisions to support health [9]. In the context of work ability literacy, this entails not only health literacy but also the knowledge, motivation and competence needed to make judgements and decisions that support work ability and to take action to promote work ability in relation to the effects and demands of work. The focus of work ability literacy varies by professional role, encompassing one’s own, clients’, or employees’ work ability.

Additionally, the concept of work-related health literacy refers to employees’ knowledge of one’s own work-related risks, threats, and requirements. The knowledge includes one’s own working conditions, needs, and knowing about available support inside and outside the workplace [11]. Moreover, the concept of occupational health literacy as (I) knowledge and skills to process occupational health information and to design health-appropriate work environments, and (II) willingness to participate in and assume responsibility for promoting health at the workplace [10]. The strength of individual’s work-related health literacy is its direct relation to one’s own work ability and employability [11]. The difference between work ability literacy [12] and work-related health literacy [11] seems to be delicate; there may be a small difference in the actions to promote work ability.

Following the definitions of health literacy, work-related and occupational health literacy, we further develop the concept of work ability literacy and incorporate the elements of information access, understanding of the information, motivation to use information, and applying the information into action. Through work ability literacy, a person recognizes the requirements and effects of their profession and can act in a way that strengthens work ability [10, 13]. We thus present work ability literacy as a combination of knowledge, understanding, motivation and practical choices to support and promote work ability, including the ability to recognize how the specific demands and effects of different occupations and work tasks shape the requirements for maintaining work ability. In Finland, a manager is a representative of the employer, with various responsibilities related to employee safety and supporting work ability [14]. While work ability literacy was originally tested among occupational health nurses, its transferability to other contexts should also be examined. Even though managers do not have equivalent skills, their responsibilities explicitly include taking care of employees’ work ability. Therefore, managers need work ability literacy to promote the work ability of both the work unit and employees. Thus, we aimed to examine HSS regarding both employees’ work ability and their own work ability. The study question was: What kind of contents does work ability literacy include according to HSS managers?

## Methods

### Data collection

The qualitative data consisted of interviews with 49 managers in HSS as part of JACARDI -project in Finland from June to October in 2024. The wellbeing services counties participating in the study informed the managers about the possibility of participating in the study through their own notification channels, and the volunteers registered through an online registration form provided by their organization. The researcher (EK) sent interview invitations to managers after receiving the contact information of the volunteer interviewees from the contact persons of the organizations. The interviewees were chosen in the order their contact information was obtained from the organizations’ contacts. The researchers didn’t set any prior criteria for the interviewees regarding work experience, unit size, or similar, so that there would be different kinds of managers among the interviewees. The interview invitation included questions about education, years of work experience as a manager and the number of employees. The background information of the interviewees is presented in Table 1.

**TABLE 1:** The background information of the interviewees (JACARDI-research project Finland, 2024).

The background information	N = 49
Organization in social welfare and healthcare	
Wellbeing services county 1	37
Wellbeing services county 2	6
Wellbeing services county 3	6
The number of employees to be managed	
Under 10 employees	4
10–19 employees	4
20–29 employees	11
30–39 employees	12
40–49 employees	9
50 or over 50 employees	9
Management education	
Yes	20
No	29
Years of work experience as a manager	
Under 5 years	17
5–9 years	9
10–15 years	12
16–20 years	6
Over 20 years	5
Gender	
Men	3
Woman	46

The interviews were conducted via Teams by four researchers (EK, NN, MK, TK). The interview framework guided the interview, but the interviewee was free to express their own views. The interviewer ensured that all planned themes were covered during the interview. The thematic interview is guided by main themes, but it allows interaction between the interviewee and the interviewer and additional questions [15]. The themes were: 1) Work ability literacy 2) Organizational guidelines and practices to support work ability 3) The managers role in supporting work ability 4) The managers's competence in supporting work ability and 5) Taking care of the manager’s own work ability. Interviews (40–105 min) were recorded and transcribed; transcripts ranged from 10 to 23 pages (Verdana 11, line spacing 1.15), totaling 740 pages.

### Data analysis

The interview data were analyzed by two researchers (EK, MP). The analysis was carried out as a deductive content analysis [16, 17]. Categorization framework for deductive content analysis was constructed based on four key dimensions of work ability literacy: knowledge, understanding, motivation and practical choices to support work ability. The researchers read the transcriptions to form an overall view. Original utterances related to the categories in the framework were extracted from the data to Excel. The coding was performed manually. Original utterances with similar content were formed into subcategories, which were named to describe their content. The researchers discussed the analysis at different stages to ensure the consistency of the categorization. All categories and examples of the original utterances are presented in Table 2.

**TABLE 2:** The main categories, subcategories and examples of the original utterances in a qualitative study on work ability literacy among managers in social welfare and healthcare (JACARDI-research project Finland 2026).

Main category	Subcategory	Original utterance
Knowledge	Organizational activities	M2: … I Get support from occupational health services; I also receive excellent support from the workplace wellbeing planner
		M30: …the truth is that the options are quite limited…, it’s about what you notice yourself, what you understand, what you come up with. Do you come up with anything? That’s probably the case in many organizations, that we don't have clear guidelines or anything like that
		M24: …we have a good amount of electronic material and training material, including workplace wellbeing programs and work ability management. At the organizational level, information and assistance are available, in my opinion, quite sufficiently. However, at the organizational level, there could perhaps be a bit more concrete practical lectures and actual tools on implementing them
	Manager’s work experience and education	M15: At least that’s how it has accumulated over the years, and the fact that you know how to find those ways to support work ability better is certainly one of them. (…) When you have started and had not yet done administrative work at all, nor the kind of manager work even though you were a team leader, they were quite strange at the time…
		M43: Through professional education, leadership studies, and then various workplace wellbeing workshops… Lectures and training days…the training I’ve attended has in some way touched upon wellbeing, … supporting wellbeing, and an understanding of what all contributes to wellbeing
	Interaction with the work community	M43: Managing that field is really challenging, and let’s say that on the public sector, far too little attention is paid to it. If we truly wanted to lead the wellbeing of subordinates, no one would have more than 25 people under them. And now it might be over a hundred, so you don't even necessarily get to see everyone… let alone have enough conversation to find out how they’re really doing, what’s going on, or what’s bothering them
		M14: First of all, quite concretely, by paying attention to what my colleagues communicate to me, I’m all ears for any indication that something might not be going well… I Maintain a close relationship with the employees by talking with everyone daily and asking how their work is going. I Pay attention to non-verbal cues as well, such as whether someone seems tired or has had many absences in a short period. I try to keep my antennae up for everyone in that way
Motivation	Ensuring the operation of the organization	M28: In my opinion, the values of the organization could be changed so that wellbeing at work would be the number one priority, because if employees are not feeling well, productivity is not very good either
		M1: Well, supporting work ability is indeed a very important matter for a manager because, of course, without employees, no operations or services can be carried out. It’s extremely important, and that’s why it’s good for a manager to know how to support employees’ work ability, their ability to cope at work, and their success in their roles
	Fulfilling the responsibilities of a manager	M20: I think that it’s just part of my job. That it’s my responsibility to take care of my employees
		M29: I Do think it’s one of the most important tasks…the main task of a manager is to allocate work and lead it in such a way that employees can and are able to sustain it in the long run
		M5: I Do have quite a lot of influence on the work community and on addressing any problems that arise…I have to address them, and they are my responsibility
		M10: …Ultimately, the manager doesn't bear responsibility for the choices an employee makes. It’s really their own matter
	Supporting the wellbeing of employees	M30: …If the work is properly organized and the employee’s resources, skills, tools, and systems are all appropriately aligned, then work ability also involves having the energy and strength to do something other than just work. Work ability should not be so narrowly tied to the job that a person can't manage anything other than performing their work tasks. Work shouldn't be one’s entire life…
		M22: … and from individual’s personal perspective as well, it is really difficult if they find themselves unable to be at work. So, it [supporting work ability] is a really important thing
Understanding	Individual characteristics	M8: Of course, there’s nothing you can do if some people have weaker starting points in life, like if their body can’t withstand the work strain and such
		M25: Well, briefly and concisely, it’s really about physical and mental work ability. From the manager’s perspective, I see that the employer has expectations for the employee’s work contribution, and if they meet those expectations, then their work ability is good
	Adaptation and modification of work	M12: Then at some point, you hit the limit of how much flexibility can be offered in this unit. You have to consider whether it might be better to think about working in another unit where the job description could be tailored
		M4: …that personal life strains on the employee. Then we’ve been able to make those decisions, short-term, temporary ones. Like reducing the workload. It hasn't required them to be completely away from work, but rather that they can partially work, or that attention has been paid to certain things. Or easing the return to work, or something like that
	A holistic view of work ability	M1: It is very important to understand what factors affect wellbeing and work ability, meaning that the job description, work objectives, and purpose should be clearly defined. Additionally, the work should be organized in such a way that aspects like client assignments and other responsibilities are distributed fairly among employees
		M31: Well, work ability is certainly about how a person copes at work, but it also involves having the resources to do the job for which the employment contract was made, for example. It includes many factors, and it’s certainly influenced by what a person does in their free time and what is happening in their personal life, but it’s also affected by what happens at work, the work environment, and the work atmosphere. Management also has an impact, for instance, if someone likes working morning shifts, whether there are plenty of those in the schedule or if there are other shifts instead
Practical choices	Modifying and managing the content of work	M27: … I’ve had those work ability negotiations and thought about the job description, 'can it be lightened somehow or if there are a few of these’ who are already part-time retired
		M29. In a way, it’s only when your work ability has already decreased that we start to consider options like part-time work or reducing the workload
	Referring employees to occupational health services	M4: We definitely need faster response times. It’s stuck; employees can't get appointments with occupational health, and it takes a long time. I Would think that accessing a work psychologist could often be a light solution for some. But even there, there can be waits of months, even half a year
		M31: We do have those proactive discussions, like if there are a lot of sick leaves or if feedback comes in from others or from a resident that someone is behaving inappropriately, then that’s exactly the time for these proactive measures. And then there’s the contact with occupational health services; I’ve been in touch with them at a low threshold, and I feel that the collaboration is quite good
	Social support	M10: But trust, it’s honestly a really big thing. It takes you a long way with the staff and with workplace wellbeing because confidentiality allows for immediate discussions and joint problem-solving, considering what’s possible and what’s not…
		M15: Well, the role is precisely to try to lead workplace wellbeing by example, and since I personally see the work atmosphere as a very strong part of it and encourage open discussion. Of course, I try to show through my own role how it works. We talk and smile, greet each other, and are friendly to one another. That’s how we strongly improve workplace wellbeing
		M12: …Discussing these kinds of things with the employee and just listening and understanding, that alone helps quite a bit. It doesn't necessarily have to be anything more than that; often, it’s enough for someone to feel heard and understood
	Enhancing employees’ professional skills	M48: … that you have been able to apply for and train in some area that interests you, which in my opinion then affects your ability to work. That it doesn't necessarily take away the osteoarthritis, but it does bring something there that then has a healing effect on the ability to work
		M38: I Discuss their hopes and thoughts, for example, regarding career planning. Personal development discussions are important in this matter, and I’ve noticed that for many, further education provides energy. Even though studying at the same time might seem demanding, the strong motivation to complete a degree is highly significant
	Promoting healthy lifestyles and recovery from work	M27: Still, you must try to maintain that physical condition and that also helps you recover in a way, of course. Adequate sleep and… Other like this…
		M38: It is very important that one eats regularly, gets enough rest, has some hobbies and so on

### Ethical considerations

Participants received oral and written information about the study, were informed that participation was voluntary, and could withdraw at any time. The results are reported in a way that ensures participants’ anonymity. The study was reviewed and approved by the ethical board of Finnish Institute of Occupational Health that has given the study a favorable statement (statement ID 154683, 22.3.2024) and have been performed in accordance with the ethical standards. As approved by the ethics committee, written informed consent was not required. Instead, all participants provided oral informed consent at the beginning of the recorded interview.

## Results

Managers’ knowledge about work ability and its promotion is shaped by organizational information, personal experience and education, and interactions within the work community. Their aim in supporting work ability is to ensure effective organizational operations, promote employee wellbeing, and fulfil their managerial responsibilities. Practical methods to support work ability include adjusting work content, referring employees to occupational health services, offering social support, enhancing professional skills, and promoting healthy lifestyles and recovery from work. We present this also in Table 1.

Managers’ work ability literacy was fragmented and uneven. Their understanding of work ability varied and was sometimes contradictory. Although managers were committed to promoting employees’ work ability, this did not extend to their own. Work ability literacy in their own work included recognition factors that influence work ability, restricting workload and searching extensively for ways to maintain and improve work ability. Work ability literacy dimensions were strongly interconnected, and gaps in one area were often associated with deficiencies in others. Managers reported unclear organizational goals and relied primarily on corrective rather than preventive measures to support work ability. No substantial differences were identified between healthcare and social care settings.

### Knowledge

Managers acquired knowledge of work ability and its promotion from multiple sources, including their employer, work experience, education, and workplace interaction, with clear differences in how actively and broadly they sought information.

#### Organizational activities

Organizations had a wide range of channels to produce and disseminate information about work ability and its promotion for managers to utilize. However, there was variability in these channels and in managers’ experiences of support from the organization, both between organizations and even between units within the same organization.

Managers varied in their views on policy usability, with many lacking the ability, skills, or opportunities to apply them effectively. All organizations had established work ability guidelines and managers utilized the specialized expertise of occupational health services and human resources. Some organizations had job wellbeing units, where wellbeing planners helped managers. Managers’ opportunities to rely on colleagues and their superiors varied.

Organizations provided quantitative and qualitative work ability indicators (e.g., wellbeing surveys, workplace assessments, and occupational health examinations), yet managers mainly relied on sickness absence alerts. Recent organizational reform clarified practices for some managers, while others reported ongoing confusion due to unrevised processes.

#### Manager’s work experience and education

Managers emphasized the know-how gained through their own work experience, or “learning the hard way.” Practical experience provided skills in engaging with employees and interpreting their work ability. It also enhanced managers’ self-efficacy to handle tasks related to work ability or sick leave. Managers’ experiences varied in how they felt their education had prepared them to support work ability. Their educational backgrounds were quite diverse, but they described the aspects of work ability literacy similarly. Regardless of the educational background, it appeared that those whose views on work ability and its support aligned with the broader understanding of work ability literacy more clearly described further training important for themselves.

#### Interaction with the work community

Managers viewed interaction within the work community as an important means of supporting employees’ work ability. Trusting relationships, informal and formal discussions, personal observation, and listening were considered key sources of information on employees’ work ability. Opportunities for day-to-day interaction varied according to team size, with some managers reporting limited personal contact due to large numbers of subordinates. Managers also noted that colleagues could raise concerns about an employee’s wellbeing or work performance. Continuous observation and attentive listening were seen as important for identifying and anticipating changes in work ability.

### Motivation

Managers were motivated to support employees’ work ability to ensure organizational operations, promote wellbeing, and fulfill their role responsibilities. Some managers argued that work ability is primarily the individual’s responsibility.

#### Ensuring the operation of the organization

Managers linked supporting work ability to ensuring organizational operations and enabling employees to perform core tasks effectively. Managers highlighted the costs due to absences and reduced work ability. Promoting work ability was recognized as a key strategy for commitment, and collaboration within the work community and preventing absences and staff turnover. However, some expressed feeling conflicted because upper management’s decision-making and policies did not consistently take employees’ work ability into consideration. The importance of supporting work ability to ensure the quality of care or patient safety was not mentioned.

#### Fulfilling the responsibilities of a manager

Managers justified supporting work ability as a duty. Many managers felt that supporting work ability was the most important part of their job. They highlighted their legal responsibility as representatives of the employer to ensure employee safety and wellbeing. However, there was uncertainty about what exactly falls under the managers’ responsibilities. Some felt a conflict between the obligation to support work ability and the available means to do so. Overall, organizational support structures for managers appeared to be unclear. Managers also pointed out that they are not entitled to information about an employee’s health unless the employee chooses to disclose it. Most managers highlighted shared responsibility for work ability, while some placed it entirely on employees.

#### Supporting the wellbeing of employees

Supporting work ability was also justified by the desire to do good for employees.

Most managers believed that the distinction between an individual’s work ability and everyday functional ability was somewhat artificial and questioned assessing employees’ wellbeing just from vocational perspective. Therefore, their aim was not only to ensure that employees had the capacity to perform their jobs, but also to make sure that work did not 'drain them empty.’ Managers considered employees as individuals with needs and obligations beyond their work hours. This perspective was often raised when discussing the benefits of autonomous shift planning.

### Understanding

Managers understood work ability and the factors influencing it heterogeneously. Some emphasized individual characteristics and the importance of private life, some the adaptation and modification of work. A holistic understanding was also mentioned.

#### Individual characteristics

Some managers perceived work ability primarily as an individual attribute, highlighting physical and mental health, professional and social skills, and motivation to learn. In this view, the significance of an employee’s private life and lifestyles such as exercise, sleep, recovery, and nutrition were highlighted. Several managers stressed that problems with work ability highlight young employees’ lack of life management and psychological flexibility. This narrow understanding of work ability was clearly associated in the data with deficiencies in other aspects of work ability literacy. Managers who regarded work ability mainly as an individual trait often did not recognize supporting work ability as part of a manager’s role, instead shifting the responsibility to the employees themselves. These managers also identified a few means at their disposal to influence their employees’ work ability.

#### Adaptation and modification of work

Most managers saw work ability as influenced by individual, life, and work-related factors. They emphasized balancing personal resources with job demands and adapting work when needed. When asked how work affects health, many perspectives arose. Most recognized that stressors are partly dependent on job description and were able to describe various stressors. However, managers perceived the impact of work and the possibilities to influence work stressors differently. Managers knew that work demand good health and specific abilities, and not all work ability issues could be resolved by adjusting work stressors. Managers described often modifying work as a solution instead of talking about supporting work ability and to take action to promote work ability in relation to the effects and demands of work before modification is needed.

#### Holistic view of work ability

Managers also considered work ability holistically. They described individual personal characteristics, and work-related stressors influence work ability. They also recognized that work ability can be maintained, enhanced, and created through organizing and managing work. Managers stated that proper orientation, a safe environment, shared rules, and skilled staff are essential for fostering work ability. Managers with a holistic understanding emphasized that workplaces need to establish structures that support work ability. In addition to actively and proactively monitoring employee wellbeing, managers should regularly review and develop work from the perspective of work ability.

Managers spoke about their own work ability in different terms than about their employees’ work ability. Even managers with a holistic view of work ability saw leadership and the work environment as irrelevant to their own work ability, considering it a personal responsibility.

### Practical choices

Supporting work ability involved modifying work content, directing employees to occupational health services, providing support, improving professional skills, and promoting healthy lifestyles and work recovery. Managers mostly described actions aimed at individual employees but also mentioned some strategies that targeted the entire work community. Corrective measures for diminished work ability were strongly emphasized.

#### Modifying and managing the content of work

Employees’ job wellbeing promotion comprised shift planning, reducing working hours, and limiting shift work. Some described these as only means of influencing employees’ work ability. In some work units, it was also possible to modify or lighten job content, such as shifting from physically demanding care tasks to lighter office work. Supporting work ergonomics was one way to enhance physical functioning at work. Managers recognizing the impact of work demands on work ability were more likely to enhance it by managing work condition. This included securing sufficient resources, limiting patient slots, and clarifying work objectives and shared rules. Some noted that time constraints and limited financial resources restricted their ability to alter team working conditions.

#### Referring employees to occupational health services

Most managers referred employees to occupational health services when work ability concerns arose, while only a few mentioned work supervision as a support measure. Experiences of collaboration with occupational health services varied considerably, ranging from smooth and valuable cooperation to perceptions of limited accessibility and long waiting times. Managers often described these actions as proactive even when work ability had already declined, suggesting that proactivity was primarily understood as preventing prolonged sickness absence or permanent work disability.

#### Social support

Providing social support—through trust, approachability, and presence—was managers’ primary proactive means of promoting work ability. Beyond gathering information on work ability, this aimed to provide emotional support, as listening and showing understanding could help prevent sick leave. Beyond listening, some managers highlighted practical problem-solving, while responsibility for employees’ wellbeing often rested on individual managerial discretion. Some managers clearly struggled to balance between the desire to support their teams and the need to maintain professional boundaries to protect their own work ability. Promoting a positive atmosphere in the work community, fostering collaboration among employees, and setting a good example were also mentioned. Despite valuing social support for subordinates, few managers mentioned it in relation to their own work ability.

#### Enhancing employees’ professional skills

Although professional competence was considered a key aspect of work ability, only a few managers described supporting their subordinates’ work ability by strengthening their skills or expertise. Some managers described organizing training, ergonomics guidance and to encourage peer learning or new responsibilities that allowed them to further develop their skills. Supporting professional skills was viewed as promoting work ability by improving occupational safety, increasing success, enhancing control to reduce stress, and making work more rewarding.

#### Promoting healthy lifestyles and recovery from work

Managers emphasized the importance of healthy lifestyles (dietary habits, exercise, adequate recovery and sleep) in maintaining work ability. However, most managers did not talk about their own ability to influence their subordinates’ lifestyles. Some encouraged healthy habits, while others saw it as impossible and intrusive into employees’ private lives.

In contrast, when discussing their own work ability, managers focused almost exclusively on maintaining healthy lifestyles during their free time. Many emphasized limiting workload as crucial for recovery, meaning not bringing work home and sticking to vacation times.

## Discussion

This study aimed to deepen the understanding of the contents of work ability literacy among HSS managers. We presented work ability literacy as a combination of knowledge, understanding, motivation and practical choices to support and promote work ability, including the ability to recognize how the specific demands and effects of different occupations and work tasks shape the requirements for maintaining work ability. Managers’ understanding of work ability is influenced by organizational and personal factors. They support work ability through work modifications, occupational health referrals, social support, competence development, and health-promoting practices, while balancing organizational needs, employee wellbeing, and managerial responsibilities.

The importance of this study is twofold. First, it shows the content and meaning of work ability literacy concept for managers of HSS organizations. The findings emphasized the manager’s role as a promoter of work ability as part of leadership and management, while the significance of work demands and their effects received less attention, despite being a key dimension of the concept of work ability literacy. Although managers were able to identify factors that contribute to employees’ work-related strain, this awareness did not always translate into preventive actions to support work ability. Strengthening this understanding would help managers adopt a more comprehensive approach to work ability support. This may also explain the differences in how they described support for their own work ability and that of their employees.

Second, the results describe many challenges related to managers’ work environment, for example variability in information, guidelines and support from organization, usability of these information and guidelines, variability in number of employees, managers experience and education. To shift work ability support from individual-focused, corrective measures toward preventive support at the work community level, organizations also need to recognize the importance of work demands and their effects on work ability. In addition, managers should be supported at the strategic level in implementing preventive approaches to the promotion of work ability. Managers require adequate resources, organizational support, education and training to effectively promote work ability, which is key to strengthening work ability management and addressing workforce shortages in HSS. The different dimensions of work ability literacy were closely intertwined. This was evident in managers’ knowledge gaps and narrow understanding of work ability, affecting motivation and decision-making. It’s concerning that even when managers were competent and committed to supporting employees’ work ability, viewing it holistically, they addressed their own work ability only through personal actions. To our knowledge, no previous studies directly support this observation. Seppänen et al. [12] observed, that occupational health nurses also typically understood work ability literacy as a combination of health, personal attributes, and life situation. From this perspective, it is not particularly surprising that this emphasis is also reflected in the understanding of HSS managers, especially as they are not required to have extensive training in work ability management. However, this does not explain the gap or fragmentation in managers’ understanding in cases where they described a holistic understanding when discussing employees’ work ability but addressed the promotion of their own work ability solely through their own actions.

The observed fragmentation of work ability literacy is linked to our finding that information gathering and practical choices focused on individual work ability and corrective actions after problems arose. Despite the managers understanding work ability support as an important task, they had very limited preventive support at their disposal. Managers’ preventive strategies that targeted the entire work community were mainly social support and interaction, which indeed have been found to support work ability [18]. Nevertheless, it is concerning if managers lack other means to promote their employees’ work ability in practice, especially when some managers already had so many employees that interacting with them personally proved almost impossible.

Our results on organizational structures, the differences of available support systems and materials across organizations, also raise concerns whether employers’ administration and higher leadership have adequate knowledge and understanding of work ability and its strategic management. Previous studies have also pointed out how dependent work ability support measures are on the organizational structures [19] and management [20]. Work ability management has been described by HSS managers as reactive, occurring in situations where there already were challenges in work ability, even though the managers themselves considered the aims of work ability management to be at a strategic level. Work ability management should combine proactive actions with reactive measures, involving both individual and organizational efforts, and aim for strategic-level goals [20]. The emphasis on corrective actions aimed at individual employees could be partly explained by the stronger evidence supporting the effectiveness of individual-level measures in enhancing work ability, as opposed to workplace-level measures. The impact of workplace and societal factors on work ability has also been studied significantly less than measures targeting individuals [21]. Assessment tools primarily emphasize individual impairments and work disability, shifting the focus from wellbeing promotion to managing reduced work ability [21, 22].

Whether challenges in work disability prevention are due to managers’ lack of competence or due to external constraints remains unclear. Organizational changes, labor shortages, and resource scarcity may hinder the effectiveness of work ability support. As conclusion we present that to strengthen managers’ work ability literacy, these factors must be resolved. Sufficiency of the workforce in HSS is an urgent challenge globally. Preventive support for work ability plays a crucial role in extending working lives and maintaining an adequate labour force [23, 24]. To sustain the work ability of the current and future workforce, improved work ability literacy and sufficient resources are necessary. Future studies should identify ways to enhance managers’ work ability literacy and support, while addressing barriers. Ensuring adequate resources and clearly defined goals and responsibilities is essential for preventive work ability support.

A limitation of this study is that work ability literacy is a relatively new and still evolving concept, which may have influenced data interpretation. We therefore sought to clearly define the concept and deepen understanding of its dimensions. The findings should also be interpreted in the context of the Finnish public social and healthcare sector, where the study was conducted. Further qualitative and quantitative research is needed across different settings.

The strength of the study is extensive interview data, which provides deep understanding of the phenomenon under study. The deductive content analysis of the data was well suited and has been carried out by two researchers [17]. study’s strength lies also in its broad view of work ability literacy, providing a new framework for describing managerial competence. Although further theoretical development is needed, the findings highlight its practical relevance and usefulness for future research.

Supporting employees’ work ability constitutes a significant part of managers’ responsibilities, requiring adequate knowledge and professional competence. The findings of this study indicate that managers’ work ability literacy, as a component of work ability support, varied considerably, and that greater attention should be paid to understanding work-related demands and their effects when supporting employees’ work ability. Therefore, further development of managers’ knowledge and competencies in this area is needed to shift the focus from individual-oriented corrective actions towards preventive measures at the work community and organizational levels. Such a transition may contribute to improving work ability, extending working careers, preventing work disability, and enhancing productivity [25].
